# A rare case report of lead encephalopathy due to high blood lead level

**DOI:** 10.1002/ccr3.7663

**Published:** 2023-07-05

**Authors:** Farnoosh Masbough, Shahin Shadnia, Mitra Rahimi, Soheil Roshanzamiri, Peyman Erfantalab Evini, Babak Mostafazadeh

**Affiliations:** ^1^ Department of Clinical Pharmacy, School of Pharmacy Shahid Beheshti University of Medical Sciences Tehran Iran; ^2^ Toxicological Research Center, Excellence Center & Department of Clinical Toxicology, School of Medicine Shahid Beheshti University of Medical Sciences Tehran Iran

**Keywords:** chelation therapy, encephalopathy, lead poisoning, neurotoxicity

## Abstract

Here we report a case of lead poisoning having a serum lead level of 412 mcg dL^−1^ who presented with decreasing level of consciousness and recurrent seizures. He responded well to treatment with chelation therapy.

## 1 INTRODUCTION

Lead is a hazardous heavy metal present in the environment; it is a delicate, bluish‐gray metal in both inorganic and organic states.^1^

The majority of adults are exposed to lead in the workplace. Because it is nonbiodegradable, its concentration accumulates in the environment. Long‐term exposure increases the risk of toxicity. There are numerous other sources of lead exposure, including the home environment, hobbies, environmental exposure, and unintentional oral consumption of lead‐contaminated substances.^2^, ^3^ The respiratory system is a significant route of lead absorption in adults, accounting for approximately 50% of all lead absorption.^4^ Although the gastrointestinal (GI) tract is not the primary route of absorption for adults, it can play a significant role, especially for those who work or eat in lead‐contaminated environments.^5^, ^6^

The kidneys remove lead from the blood relatively quickly, with a half‐life of 30 to 40 days in adults and more prolonged in children and pregnant women.^7^ Chronic lead exposure below 10 μg dL^−1^ is associated with health risks. Blood lead level (BLL) remains the gold standard for determining lead exposure.

BLL levels greater than 80 μg dL^−1^ are most likely to cause symptoms. When BLL is between 40 and 80 μg dL^−1^, symptoms are less severe and manifest to a more variable degree. Adults with BLL concentrations below 40 μg dL^−1^ are frequently asymptomatic; therefore, physicians should investigate alternative causes for symptoms.

Depending on the amount of lead ingested, acute lead poisoning can cause symptoms of severe toxicity or nonspecific signs and symptoms, such as neurological, hematologic, and renal system damage. GI issues (anorexia, vomiting, constipation, and abdominal pain), hypertension, and infertility may occur also.

The molecular mechanisms of lead toxicity include disrupting calcium homeostasis, inducing oxidative stress, and interfering with cellular signaling pathways.^8^

Lead toxicity can induce seizures through several mechanisms, including disruption of neurotransmitter systems, alteration of neuronal excitability, and oxidative stress.^9^

In addition, continuous exposure to moderate or even low levels of lead may not cause symptoms but increases the likelihood of long‐term health effects.

## 2 CASE PRESENTATION

**FIGURE 1:**
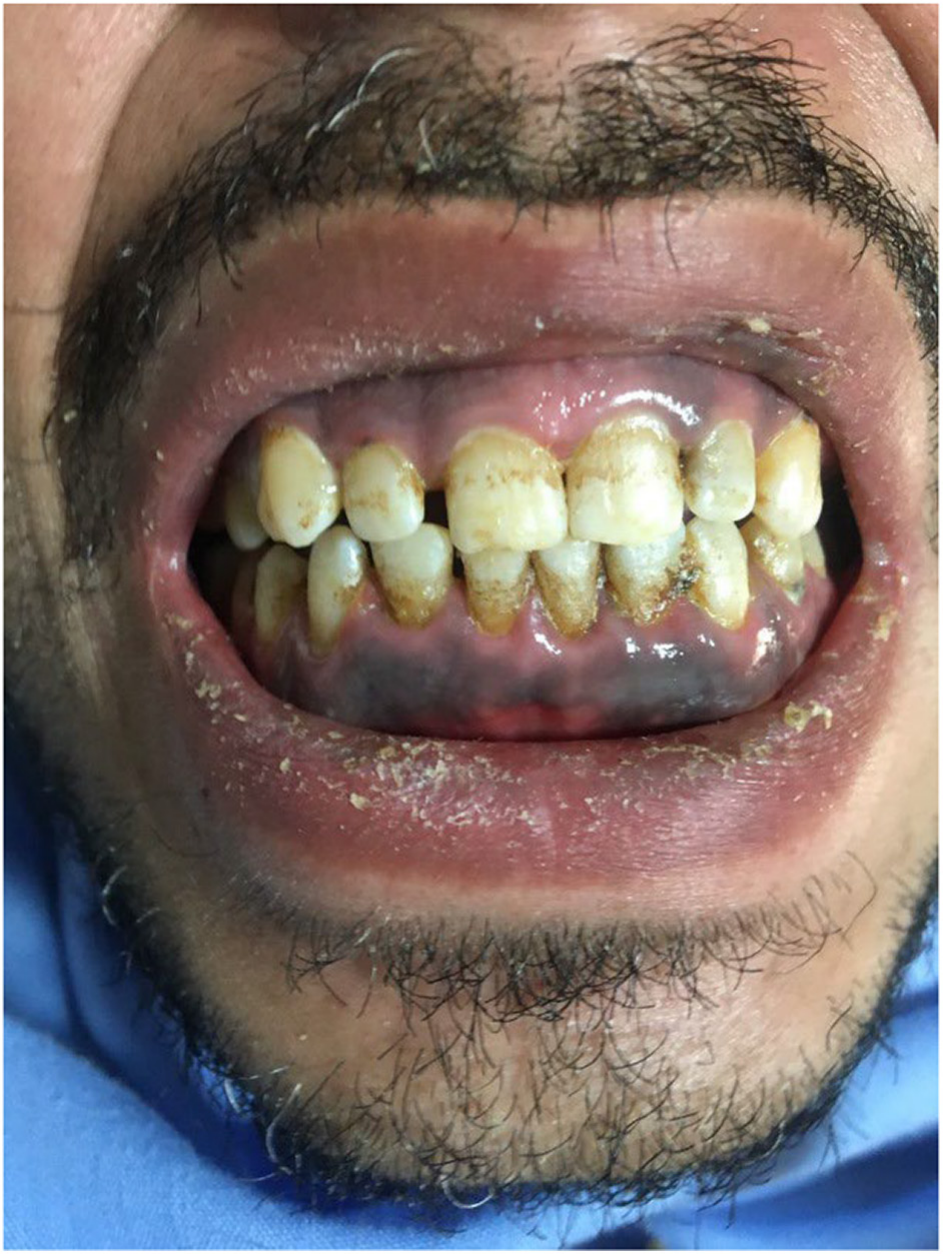
Burtonian line in gingival tooth border.

Our patient is a 27‐year‐old male who presents with a several year history of opium addiction with a two‐month history of bone pain and several episodes of seizures before being hospitalized. The bone pain was due to the involvement of multiple joints, and the patient's convulsions were episodes of generalized tonic–clonic seizure (GTC). He had been working in a machine‐manufacturing factory. The patient's medical history was negative for any disease. His vital signs were not significant for any abnormality. Due to joint pain, he underwent a general workup with a blood lead level of 412 μg dL^−1^ (normal value, <10 μg dL^−1^), and finally, he was referred to a hospital toxicology department. He was ill, drowsy, and cachectic on presentation to the emergency department. Physical examination revealed abdominal tenderness, delirium, and agitation. In addition, the patient had bilateral upper and lower limb neuropathies, which resulted in wrist drops. In the examination, except for peripheral neuropathy, there was no evidence of muscle disorders or tremors. In addition, the tooth line demonstrated lead lines and bluish discoloration of the gingiva (Figure 1). The complete blood count revealed normocytic‐hypochromic anemia with normal white blood cells and platelet count. Anemia may be due to a decrease in hemoglobin production and hemolysis. Based on normal LDH and peripheral blood smear, hemolysis was removed from differential anemia diagnoses. Blood urea nitrogen, serum creatinine, electrolytes, and liver enzymes were normal. The ECG was unremarkable and cardiac enzymes were within normal limits. Imaging studies, including chest radiography, pelvic and abdominal ultrasound, and brain CT scan, did not show any significant findings.

Due to the patient's level of consciousness and history of seizures before being admitted to the hospital, he was transferred to the intensive care unit. Treatment includes chelation therapy with 3 mg kg^−1^ intramuscular dimercaprol every 4 h for 3 consecutive days and CaNa_2_ EDTA, 1000 mg m^−2^ every 8 h for 5 days. Other prescription drugs include pantoprazole 40 mg once daily and oxycodone 5 mg every 8 h. For the treatment and prophylaxis of GTC levetiracetam 500 mg every 12 h, and diazepam 10 mg intravenous standby, was initiated. On the third day of hospitalization, the patient's consciousness and oxygen saturation level decreased, requiring endotracheal intubation. He did not experience any further seizure activity. The brain CT scan revealed no abnormalities, but the electroencephalogram (EEG) showed mild generalized encephalopathy. After 72 h of intubation, the patient had a temperature of 38.2°C, a heart rate of 100 beats per minute, a blood pressure of 125/80 mmHg, and oxygen saturation of 88%. Blood culture, urine culture, and sputum culture were performed. Upon physical examination, breath sounds were absent in both lungs, and the chest x‐ray revealed bilateral opacities with massive pleural effusion. Empiric antibiotics, including vancomycin 1000 mg twice daily intravenously, meropenem 1000 mg intravenously three times a day, and ciprofloxacin 400 mg intravenously three times a day, are started when the patient is suspected of having ventilator‐associated pneumonia. The blood lead level was 35 μg dL^−1^, and the course of all chelating therapy was over, and then succimer (2,3 dimercaptosuccinic acid) 30 mg kg^−1^ by mouth for 5 days followed by 20 mg kg^−1^ for a further 14 days was started. Blood and urine cultures were negative. The reason for stopping vancomycin and ciprofloxacin was Pseudomonas sputum culture (10^5^ colony‐forming unit). meropenem was continued for 14 days. Two days after the antibiotic was administered, the patient's fever subsided, and oxygen saturation reached 94%. He was extubated 1 week after intubation due to increased consciousness and improved lung function. The patient was then transferred to the general ward after extubation. The patient's response to chelator therapy was remarkable. Five days later, his blood lead level was 25 μg dL^−1^, and he was discharged from the hospital in good condition.

## 3 DISCUSSION AND CONCLUSION

In this case study, we describe a rare case of lead toxicity that caused bone pain, seizures, and abdominal pain. As far as we searched in the articles, chronic lead toxicity has never been reported, with a serum level of 412 μg dL^−1^ at admission. Although there is evidence that chronic lead exposure below 10 μg dL^−1^ may carry risks, other studies on chronic lead poisoning mean BLL 10 μg dL^−1^ is dangerous to life or health.^10^ In cases of opium addiction, simultaneous anemia and pain have been reportable in the last 10 years. There is lead poisoning in Iran with opium impurities and lead‐contaminated opium.^11^, ^12^ Because there was no mortality despite the highly high BLL level (412 μg dL^−1^), our case is exceptional.

Beigmohammadi et al. reported a case of an irreversible motor neuron defect in an opium‐dependent patient with BLL > 200 μg dL^−1^.^13^ In the second study, a man with a history of schizophrenia experienced acute lead poisoning after ingesting 22‐caliber lead bullets, which resulted in severe lead poisoning (BLL 391 μg dL^−1^). After aggressive GI decontamination and chelation therapy, he recovered.^14^

Burtonian or “lead line,” a rare sign of chronic lead poisoning, was present in this patient. It is a bluish gingival pigmentation at the gum‐tooth line caused by a reaction between lead and bacteria in dental plaque that results in the formation of lead sulfide. According to a case study by Banagozar Mohammadiet al., a 32‐year‐old factory worker had a BLL of 165 μg dL^−1^ presented with a Burtonian at the gum‐tooth line.^15^ Our patient's typical symptoms of lead toxicity included delirium, agitation, and pain in the bones and abdomen.

After admission, we immediately began chelation therapy and lead‐related health effects assessment because the BLL was over 100 μg dL^−1^. It was influential in the treatment of dimercaprol and calcium disodium edetate. Adding lead to opium is a common occurrence in Iran.

In this case, the source of the intoxication was likely from oral opium consumption. Since the clinical manifestations of lead poisoning are nonspecific and can be misdiagnosed with other pathophysiologic conditions, physicians should be advised and assess the potential for lead poisoning in opium users.

Chronic opium users with symptoms like abdominal pain, weakness, lethargy, anemia, convulsions, or other neurological and psychological symptoms should be evaluated for lead poisoning despite its many causes. Early detection of lead poisoning is crucial because it allows treatment to begin before permanent damage to the nervous system occurs. Lead poisoning and encephalopathy are challenging to treat, but chelators, especially the injectable form, may help.

## AUTHOR CONTRIBUTIONS

**Farnoosh Masbough:** Conceptualization. **Shahin Shadnia:** Methodology; supervision. **Mitra Rahimi:** Conceptualization; supervision. **Soheil Roshanzamiri:** Writing – original draft. **Peyman Erfantalab Evini:** Methodology; writing – original draft. **Babak Mostafazadeh:** Conceptualization; writing – original draft; writing – review and editing.

## FUNDING INFORMATION

No additional funding for the execution of the present study was received.

## CONFLICT OF INTEREST STATEMENT

The authors declare that they have no competing or conflict of interests.

## CONSENT

Written informed consent had been obtained from the patient to publish this report in accordance with the journal's patient consent policy.

## Data Availability

The datasets generated during the current study are available and accessible. Additional data related to this study, including any supplementary materials, can be obtained from the corresponding author upon reasonable request.
